# Utility of Host Markers Detected in Quantiferon Supernatants for the Diagnosis of Tuberculosis in Children in a High-Burden Setting

**DOI:** 10.1371/journal.pone.0064226

**Published:** 2013-05-15

**Authors:** Novel N. Chegou, Anne K. Detjen, Lani Thiart, Elisabetta Walters, Anna M. Mandalakas, Anneke C. Hesseling, Gerhard Walzl

**Affiliations:** 1 DST/NRF Centre of Excellence for Biomedical Tuberculosis Research and MRC Centre for Molecular and Cellular Biology, Division of Molecular Biology and Human Genetics, Department of Biomedical Sciences, Faculty of Medicine and Health Sciences, Stellenbosch University, Tygerberg, South Africa; 2 Desmond Tutu TB Centre, Department of Paediatrics and Child Health, Faculty of Medicine and Health Sciences, Stellenbosch University, Tygerberg, South Africa; 3 The International Union Against Tuberculosis and Lung Disease, North America Office, International Union Against Tuberculosis and Lung Disease, New York, New York, United States of America; 4 Section on Retrovirology and Global Health, Department of Pediatrics, Baylor College of Medicine, Houston, Texas, United States of America; McGill University, Canada

## Abstract

**Background:**

The diagnosis of childhood tuberculosis (TB) disease remains a challenge especially in young and HIV-infected children. Recent studies have identified potential host markers which, when measured in Quantiferon (QFT-IT) supernatants, show promise in discriminating between *Mycobacterium tuberculosis (M.tb)* infection states. In this study, the utility of such markers was investigated in children screened for TB in a setting with high TB incidence.

**Methodology and Principal Findings:**

76 children (29% HIV-infected) with or without active TB provided blood specimens collected directly into QFT-IT tubes. After overnight incubation, culture supernatants were harvested, aliquoted and frozen for future immunological research purposes. Subsequently, the levels of 12 host markers previously identified as potential TB diagnostic markers were evaluated in these supernatants for their ability to discriminate between *M.tb* infection and disease states using the Luminex platform. Of the 76 children included, 19 (25%) had culture confirmed TB disease; 26 (46%) of the 57 without TB had positive markers of *M.tb* infection defined by a positive QFT-IT test. The potentially most useful analytes for diagnosing TB disease included IFN-α2, IL-1Ra, sCD40L and VEGF and the most useful markers for discriminating between QFT-IT positive children as TB or latent infection included IL-1Ra, IP-10 and VEGF. When markers were used in combinations of four, 84% of all children were accurately classified into their respective groups (TB disease or no TB), after leave-one-out cross validation.

**Conclusions:**

Measurement of the levels of IFN-α2, IL-1Ra, sCD40L, IP-10 and VEGF in QFT-IT supernatants may be a useful method for diagnosing TB disease and differentiating between active TB disease and *M.tb* infection in children. Our observations warrant further investigation in larger well-characterized clinical cohorts.

## Introduction

Tuberculosis (TB) remains a global health problem and the diagnosis remains challenging especially in children, who typically develop paucibacillary disease [1]. The introduction of the XpertMTB/RIF assay (Cepheid Inc., CA, USA) into routine clinical practice [2] is a significant improvement especially in high-burden settings since diagnosis of pulmonary TB is now possible within 2 hours, coupled with the detection of rifampicine resistance [3]. However, many limitations including the high operating costs [4] are major impediments to large-scale roll-out of such tests in resource-limited settings. Furthermore, sputum based tests have limitations for the detection of *Mycobacterium tuberculosis (M.tb)* in children, both due to the low organism yield, and limited tussive force. In children hospitalized for suspected pulmonary TB and with radiological evidence of disease on chest radiograph, only 30–40% are culture-confirmed if sampled repeatedly by gastric aspiration, nasopharyngeal aspiration or sputum induction [5]. Furthermore, culture is costly and results may take up to 6 weeks [6]. There is a need for new, rapid and accurate diagnostic tools more effective in detecting paucibacillary TB in young children. Ideally, such methods should be coupled with the development of suitable platforms for detection such as incorporation of validated markers into rapid point-of-care tests that are feasible to use in resource-limited settings. Such tests would ideally use readily obtainable paediatric specimens including small volumes of whole blood, serum/plasma, saliva, stool or urine.

Commercial Interferon gamma (IFN-γ) release assays (IGRAs) such as the ELISA-based Quantiferon TB Gold In-Tube assay (QFT-IT; Qiagen, Germany), and the ELISPOT-based T SPOT.*TB* (Oxford Immunotec, UK), are widely used especially in high income, low burden settings for the diagnosis of *M.tb* infection and for research in high-burden TB settings. These assays have proven to be useful to accurately detect *M.tb* infection in adults and children [7]–[10], but are unable to discriminate between *M.tb* infection and active TB disease [11]. Investigations aiming at identifying other potentially useful *M.tb* diagnostic antigens are on-going [12]–[15]. At the same time, the identification of additional host markers in supernatants upon stimulation with the widely investigated ESAT-6/CFP-10/TB7.7 (the latter only in QFT-IT assays) may be advantageous since new assays based on such markers could build on this existing well-developed platform [14], [16]–[19], [19]–[26]. Studies investigating such markers of infection have identified interferon inducing protein (IP)-10, macrophage chemotactic protein (MCP)-1, MCP-2, Interleukin (IL)-2, interleukin-1 receptor antagonist (IL-1Ra) and tumour necrosis factor (TNF)-α, amongst others, as potential alternative markers to IFN-γ [18], [21]–[23], [25], [27]. Other studies have indicated that epidermal growth factor (EGF), macrophage inflammatory protein (MIP)-1β, IL-1α, transforming growth factor (TGF)-α, soluble CD40 ligand (sCD40L), vascular endothelial growth factor (VEGF), IP-10, TNF-α, IL-12(p40), might discriminate between active TB and infection [16], [19], [20], [28]. The available data on the potential utility of these markers, especially in discriminating between TB disease and infection are limited, conflicting and the studies conducted to date have used highly heterogeneous methodology. In the present study, we investigated the utility of 13 markers previously reported [16], [18]–[23], [25], [27], [28] to be potentially useful in the diagnosis of *M.tb* infection or TB disease in children from a setting with high burden of TB and HIV.

## Materials and Methods

### Ethics Statement

The children included in this study were enrolled as part of two larger paediatric studies. Upon completion of initial sample collection, frozen aliquots of QFT-IT supernatants were thawed and used for the Luminex experiments presented in this report. The larger studies were approved by the Health Research Ethics Committee of Stellenbosch University (project numbers N07/08/180 and N08/08/207). Parents gave written consent for study participation, including for HIV testing and storage of blood samples for future TB diagnostic research.

### Study Participants and Setting

The two larger paediatric studies that contributed participants for this study were conducted in 2008 in Cape Town, Western Cape Province, South Africa. In Cape Town, the TB notification rate among children aged 0–13 years was 620/100 000 in 2008 [29]. BCG vaccination (Danish strain, 1331, Statens Serum Institute, Copenhagen, Denmark) is routinely administered at birth (99% coverage). Study 1 aimed to assess the value of commercial IGRAs for the diagnosis of active TB in hospitalized children with different spectrum and severity of disease (Table 1) and contributed all the 19 TB cases included in this sub-study. Study 2 was a paediatric household contact study which aimed to investigate markers of TB infection in recently TB-exposed children. All 57 children without active TB disease were recruited from study 2.

**Table 1:** Patient characteristics.

	All	Tuberculosis$	QFT-IT positive non-cases	*M.tb* uninfected	P value 1	P value 2
**N (%)**	76	19 (25)	26 (34.2)	31(40.8)	–	–
**Age, Months (IQR)**	37.6 (13.8–48.7)	36.7 (6.0–55.0)	41.4 (18.1–55.6)	35.1 (14.1–38.3)	0.552	0.223
**Males, n(%)**	42 (55.3)	13 (68.4)	10 (38.5)	19 (61.3)	0.28	0.052
HIV infected, n(%)	22(29.0)	4(21.0)	9(34.6)	9(29.0)	0.480	0.560
Quantiferon results≠						
**Positive, n (%)**	41(54.0)	15 (79.0)	26 (100.0)	0 (0.0)	0.027	0.027
**Negative, n (%)**	33 (43.0)	2 (10.5)	0 (0.0)	31 (100.0)	<0.0001	–
**Indeterminate, n(%)**	2 (2.6)	2 (10.5)	0 (0.0)	0 (0.0)	–	–

$ Two of the children had contained pulmonary TB disease (Ghon focus), 12 had uncontained pulmonary TB and 5 had disseminated TB disease. Of the 12 children with uncontained pulmonary TB, 8 had alveolar opacification with pleural effusion (n = 2) or without effusion (n = 6), 2 had expansile pneumonia (n = 1 with obstruction and collapse), and 2 had bronchopneumonic spread (n = 1 with pleural effusion). Of the 5 children with disseminated disease, 2 had miliary TB, 2 had TB meningitis and miliary TB, and 1 had spinal TB with alveolar opacification.

≠ **Positive**: Nil IFN-γ ≤8.0 IU/ml and TB Ag-Nil ≥0.35 IU/ml and ≥25% of Nil value; **Negative**: Nil IFN-γ ≤8.0 IU/ml and TB Ag-Nil <0.35 IU/ml and Mitogen-Nil ≥0.5 IU/ml, or Nil IFN-γ ≤8.0 IU/ml and TB Ag-Nil ≥0.35 IU/ml and <25% of Nil value and Mitogen-Nil ≥0.5 IU/ml; **Indeterminate:** Nil IFN-γ >8.0 IU/ml, or Nil IFN-γ <8.0 IU/ml and TB Ag-Nil <0.35 IU/ml and Mitogen-Nil <0.5 IU/mL, or Nil IFN-γ <8.0 IU/ml and TB Ag-N ≥0.35 IU/ml and <25% of Nil value and Mitogen <0.5 IU/ml.

P-value 1: Number of individuals with variable/outcome and with TB vs number with variable but without TB, P-value 2: Number of individuals with variable and with LTBI vs number with variable and with Active TB.

IQR: Interquartile range.

Study 1 (disease group) included children with symptoms and signs suggestive of TB recruited at Tygerberg Children’s Hospital; study 2 (no-disease group) enrolled children exposed to an adult with smear or culture confirmed pulmonary TB in the household. Participants in both parent studies were aged 3 months to 5 years. Children were excluded if weighing <2.5 kg and if they had received antituberculosis therapy for >1 month (disease group) or were on any antituberculosis therapy (no-disease group).

All children were systematically screened for TB through history, clinical examination, Mantoux tuberculin skin test [TST; 2 tuberculin units of purified protein derivative (PPD RT23), SSI, Denmark], chest radiography (read by two blinded experts using a standardized reporting form) [30], and liquid mycobacterial culture (MGIT system, Beckton-Dickinson, USA) of minimum 2 respiratory samples (typically gastric aspirates) and of any other clinically relevant specimens. Positive cultures were speciated using line probe assay (GenoType® MTBDR*plus*, Hain Lifescience GmbH, Nehren, Germany). HIV infection status was determined in all children (HIV DNA PCR if < = 18 months and HIV ELISA if >18 months of age). All participants provided a 3 ml whole blood sample that was collected directly into three QFT-IT tubes (Nil, mitogen and TB antigen tubes) as recommended by the manufacturer (Qiagen, Germany). The tubes were transported at room temperature to the research laboratory within 2 hours of collection and incubated (37°C, 20–24 hours); thereafter, supernatants were harvested, aliquoted and frozen at −80°C for the immunological assays described below.

Confirmed TB disease was defined as bacteriological identification of *M.tb* from any sample, in the presence of clinical and radiological signs and symptoms. TB was excluded (“unlikely” or “not” TB) [31] if the child was asymptomatic (study 1), with a chest radiograph not suggestive of TB, and negative mycobacterial cultures. Children with probable or possible TB disease were excluded from this study. Children with confirmed TB disease with available frozen QFT-IT supernatants (Study 1) were age-matched to controls without TB (Study 2), with or without *M.tb* infection, and with available supernatants.

For the purpose of this sub study, *M.tb* infection was defined as a positive QFT-IT result, in the absence of any other clinical or radiological signs and symptoms suggestive of TB or positive MGIT cultures. Laboratory analysis was blinded to all clinical data including TB infection or disease status and the clinical team was blinded to QFT-IT results.

### Immunoassays

IFN-γ responses in culture supernatants were determined using the Quantiferon TB Gold ELISA kit and the results were interpreted for *M.tb* infection using the analysis software provided by the manufacturer (Qiagen, Germany) as previously described [32]. Frozen aliquots of each participant’s unstimulated (nil) and TB antigen-stimulated supernatants were thawed and the levels of 12 host markers (EGF, IFN-α2, IL-1Ra, IL-1α, IP-10, MCP-3, MIP-1β, sCD40L, TGF-α, TNF-α, VEGF and IFN-γ) evaluated using customized Milliplex kits (Merck Millipore, St. Charles, Missouri, USA) on the Bio Plex platform (Bio Plex™, Bio Rad Laboratories) as previously described [20]. Prior to the assay, all supernatants were diluted 1∶1 with the kit serum matrix to ensure accurate measurement of chemokine levels following previous optimization experiments [20]. The concentration of IP-10 in TB antigen stimulated supernatants, however, remained above the range of the standard curve in 14/19 (74%) TB cases and 31/57(54%) non TB cases; IP-10 TB antigen data was therefore excluded from further analysis. Samples were evaluated blinded to QFT-IT status. All analyte levels in the two quality control reagents supplied by the manufacturer were within the expected ranges. Marker concentrations detected in the different supernatants were automatically multiplied by 2 (to correct for the dilution) by the software used for bead acquisition and analysis, the Bio Plex manager software, version 4.1.1. The standard curve for all biomarkers ranged from 3.2–10000 pg/ml.

### Statistical Analysis

Differences between the comparison groups (e.g. TB disease and no TB) were determined using the Mann–Whitney U test for non-parametric data analysis. Optimal cut-off levels for differentiating between groups were determined by receiver operator characteristics (ROC) curve analysis, based on the highest likelihood ratio. The predictive abilities of combinations of analytes for the different *M.tb* infection and disease states were estimated by performing best subsets general discriminant analysis (GDA), with leave-one-out cross validation [20]. Differences between groups were considered significant if p values were ≤0.05. The data were analysed using the Statistica software (Statsoft, Ohio, USA) and GraphPad Prism, version 5.00 for Windows (GraphPad Software, San Diego, CA, USA).

## Results

A total of 76 children, 19 with culture confirmed TB disease and 57 without were included. Using the manufacturer’s recommended definition for QFT-IT, with a cut-off value of 0.35 IU/ml, 15/19 (78.9%) of the children with TB and 26/57 (45.6%) of those without TB were positive for *M.tb* infection. The mean age (±SD) of study participants was 37.6±33.1 months; 22 (29%) were HIV-infected. The clinical and demographic characteristics of study participants are shown in Table 1.

### Utility of Host Markers in the Diagnosis of TB Disease

The concentrations of the markers in the supernatants harvested from the unstimulated (nil) and the TB-specific antigen QFT-IT tubes from children with culture confirmed TB disease (n = 19) were compared with the levels in the 57 without TB disease (45.6% of whom were infected). The unstimulated (N), antigen stimulated (Ag) and the antigen specific responses of each marker, obtained by subtraction of the unstimulated levels from the antigen stimulated responses (Ag-N, with the exception of IP-10), were analysed as separate variables in order to evaluate the contribution of unstimulated marker levels to the diagnosis of disease. Unstimulated IP-10 responses were also included in the analysis as these levels were within the measurable range of the standard curve (up to 20000 pg/ml taking into account the dilution factor) in all study participants. Of the 12 markers evaluated, the median unstimulated, antigen stimulated, or antigen-specific levels of seven (IFN-α2, IL-1Ra, IP-10, sCD40L, VEGF, MCP-3, IFN-γ) were either significantly different between the two groups or showed trends (Table 2): the unstimulated levels of IFN-α2 and IL-1Ra were significantly higher (p<0.05) in children without TB disease, while the median unstimulated levels of IP-10 and sCD40L were significantly higher in those with disease, with the difference in the unstimulated levels of VEGF (higher in TB) and MCP-3 (higher in non-TB) showing a trend towards significance (p = 0.06 and 0.07 respectively) (Table 2). When the TB antigen-specific marker responses were calculated by subtraction of the respective unstimulated control levels, only the median levels of VEGF and IFN-γ were significantly different between the TB and non-TB groups, with the levels of sCD40L showing a trend towards significance (0.05<p≤0.08) (Table 2). The median levels of all the markers investigated, in the children with TB disease, those with QFT-IT positive results but without TB disease, and the *M.tb* uninfected children is shown in Table S1.

**Table 2:** Median levels of analytes (pg/ml) and ranges (in parenthesis), and accuracies in the diagnosis of TB disease in all study participants.

Marker$	No TB (n = 57)	TB disease (n = 19)	P-value	AUC (95% CI)	Cut-off value	Sensitivity, % (95% CI)	Specificity,% (95% CI)
**IFN-α2_N_**	**5.8 (0.0–299.4)**	**0.0 (0.0–109.8)**	**0.005**	**0.70 (0.57–0.83)**	**<5.0**	**89.5 (66.9–98.7)**	**52.6 (39.0–66.0)**
IFN-α2_Ag_	5.8 (0.0–269.4)	0.0 (0.0–133.2)	0.012	0.68 (0.55–0.81)	<4.7	84.2 (60.4–96.6)	52.6 (39.0–66.0)
**IL-1ra_N_**	**187.9 (0.0–4991.4)**	**63.4 (0.0–717.0)**	**0.01**	**0.70 (0.55–0.84)**	**<11.8**	**26.3 (9.1–51.2)**	**98.2 (90.6–99.9)**
IP-10_N_	3967.0 (474.6–>20000)	7613.0 (1075.0–>20000)	0.03	0.66 (0.52–0.80)	>6906	63.2 (38.4–83.7)	75.4 (62.2–85.9)
**sCD40L_N_**	**4722.2 (1296.0–** **>20000)**	**7858.2 (1177.2–>20000)**	**0.0095**	**0.70 (0.55–0.84)**	**>10877**	**42.1 (20.3–66.5)**	**93.0 (83.0–98.0)**
**sCD40L_Ag_**	**3917.3 (1135.0–** **>20000)**	**9769.3 (1296.0–>20000)**	**0.0028**	**0.73 (0.58–0.89)**	**>9671**	**52.6 (28.9–75.6)**	**93.0 (83.0–98.0)**
VEGF_Ag_	**542.5 (0.0–4134.0)**	**963.7 (325.4–2637.2)**	**0.0056**	**0.71 (0.60–0.82)**	**>809.3**	**84.2 (60.4–96.6)**	**66.7 (52.9–78.6)**
VEGF_Ag-N_	**0.0 (0.0–3721.4)**	**302.4 (0–2000.0)**	**0.0065**	**0.71 (0.59–0.83)**	**>32.0**	**84.2 (60.4–96.2)**	**68.4 (54.8–80.1)**
IFN-g_Ag-N♯_	13.1 (0–8774.9)	170.4 (0.0–9383.4)	0.02	0.68 (0.55–0.81)	>2587	26.3 (9.1–51.2)	98.3 (90.6–99.9)
MCP-3_N_	3185.0 (258.3–>20000)	2085.0 (319.8–>20000)	0.08	0.63 (0.49–0.78)	<640.9	26.3 (9.1–51.2)	94.7 (85.4–98.9)
sCD40L_Ag-N_	0.0 (0.0–50.5)	1147.2 (0.0–9699.6)	0.08	0.63 (0.46–0.81)	>2977	36.8 (16.3–61.6)	94.7 (85.4–98.9)
VEGF_N_	458.2 (0.0–3966.4)	727.5 (0.0–1011.7)	0.06	0.65 (0.52–0.77)	>625.2	83.3 (58.6–96.4)	61.4(47.6–74.0)
IFN-g_Ag_	32.8 (0.0–8774.9)	170.4 (0.0–9383.4)	0.08	63 (0.48–0.78)	>2623	26.3 (9.1–51.2)	98.3 (90.6–99.9)

Participants were not stratified according to Quantiferon results or HIV status prior to analysis. All analytes that showed significant differences (P<0.05) or trends according to the Mann Whitney U test are shown. Analytes that discriminated between TB disease and no TB disease with AUC ≥0.70 after ROC analysis are highlighted in bold. Cut-off values were determined based on the highest likelihood ratio. Sensitivity and specificity are expressed as a percentage. AUC = Area under the ROC curve, 95% CI = 95% confidence interval.

$ N = unstimulated marker levels, Ag = Antigen stimulated levels, Ag-N = the difference between the antigen stimulated and the unstimulated responses; ♯At the cut-off value used in the QFT-IT test (0.35 IU/ml = 14 pg/ml [60]), the sensitivity of IFN-γ for TB disease was 79.0 (54.4–93.9) and specificity was 50.9 (37.3–64.4).

When the diagnostic accuracy of the markers was investigated by receiver operator characteristics (ROC) curve analysis, the markers that showed promise as diagnostic candidates as determined by area under the ROC curve (AUC) ≥0.70 [20], [33], included the unstimulated levels of IFN-α2, the unstimulated levels of IL-1Ra, the unstimulated levels of sCD40L, the antigen stimulated levels of sCD40L, VEGF, and the antigen-specific levels of VEGF (Table 2). When the cut-off value with the highest likelihood ratio was selected, the sensitivity of IFN-α2 (N), VEGF (Ag) and VEGF (Ag-N) were all ≥84.0%, while specificity was only between 52.6% and 68.4%. IL-1Ra (N), sCD40L (N) and sCD40L (Ag) all predicted TB disease with sensitivity ≤53% but with specificity ≥93.0% (Table 2).

When the median levels of markers obtained in the children with TB disease were compared to levels obtained in non-diseased children but restricting the analysis to HIV-uninfected children (15 children with TB disease and 39 without TB disease), significant differences or trends were observed for the unstimulated, antigen stimulated or antigen-specific levels of eight of the 12 markers investigated (IFN-α2, IL-1Ra, IP-10, sCD40L, VEGF, IFN-γ, EGF and IL-1α) (Table S2). After ROC analysis, AUC was ≥0.70 for IFN-α2 (N), IFN-α2 (Ag), IL-1Ra (N), VEGF (N), VEGF (Ag), VEGF (Ag-N) and IFN-γ (Ag-N) (Table S2). Although these markers detected TB disease with sensitivity up to 88.0% and specificity up to 97% at the cut-off values with the highest likelihood ratio, only VEGF (N, Ag and Ag-N) ascertained TB disease with both sensitivity and specificity >75% (Table S2).

**Figure 1:**
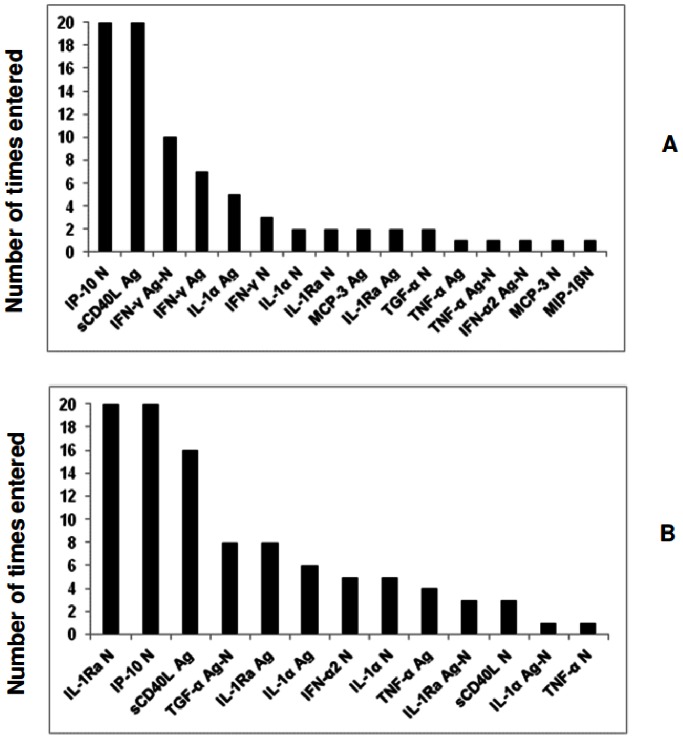
Frequency of analytes in the top 20 GDA models that most accurately classified all study participants into respective groups. Participants were not stratified according to HIV infection status or Quantiferon results prior to analysis of the data. The columns represent the number of times each analyte was included into the top 20 discriminatory models. A = frequency of analytes in the models generated with raw untrimmed data, B = frequency of analytes in models generated after data was trimmed to scale-down the influence of outliers.

To investigate whether the diagnostic accuracy of the markers could be improved if used in combination, data for all participants (irrespective of HIV status) were fitted into general discriminant analysis (GDA) models. Similarly to the univariate analysis, the unstimulated (N), antigen stimulated (Ag) and the antigen-specific responses (Ag-N) of all markers were considered as separate variables, to determine the contribution of unstimulated marker levels in models. Optimal prediction of TB or no TB disease was achieved when analytes were used in combinations of four (Table 3). Although 86% of the non TB cases could be accurately predicted when IP-10 (N), MCP-3 (Ag), sCD40L(Ag) and IFN-γ (Ag-N) responses were combined, only 68% of the TB cases could be accurately predicted by combining any four markers after leave-one-out cross validation (Table 3). When the data was trimmed using a statistical procedure in which the influence of outliers on the data is scaled down, before analysis, there was an increase in the predictive abilities of the 4-analyte models (Table S3), with the three most accurate models comprising of four-analyte combinations between IL-1Ra (N), IL-1α (N), IP-10(N), sCD40L(Ag), TNF-α(N), TGF-α (Ag-N) and IFN-α2 (Ag), accurately predicting up to 84% (48/57) of the non TB cases and up to 84.2% (16/19) of the TB cases after leave-one-out cross validation (Table S3). The most frequently occurring analytes in the top 20 GDA models that accurately classified participants as TB disease or no TB disease in the raw untrimmed data included IP-10 (N), sCD40L (Ag) and IFN-γ (Ag-N), while the most frequent analytes in the top 20 models generated from the trimmed data included IL-1Ra (N), IP-10 (N) and sCD40L (Ag) (Figure 1).

**Table 3:** General discriminant analysis (GDA) models for discriminating between TB disease and no TB in all study participants.

Analytes	Resubstitution Classification matrix			Leave-one-out Cross validation		Wilks lambda	f
	No TB (%)	TB (%)	Total (%)	No TB (%)	TB (%)		
IL-1α_Ag_ IP-10_N_ sCD40L_Ag_ IFN-γ_Ag-N_	87.7 (50/57)	57.9 (11/19)	80.3 (61/76)	82.5(47/57)	57.9 (11/19)	0.327551	145.76
IL-1α_Ag_ IP-10_N_ sCD40L_Ag_ TNF-α_Ag_	82.5 (47/57)	63.2 (12/19)	77.6 (59/76)	80.7 (46/57)	57.9 (11/19)	0.322849	148.91
IP-10_N_ MCP-3_Ag_ sCD40L_Ag_ IFN-γ_Ag-N_	87.7 (50/57)	68.4 (13/19)	82.9 (63/76)	86.0 (49/57)	63.2 (12/19)	0.3445	135.0
IP-10_N_ MCP-3_Ag_ sCD40L_Ag_ IFN-γ_Ag_	87.7 (50/57)	68.4 (13/19)	82.9 (63/76)	84.2 (48/57)	63.2 (12/19)	0.3465	133.9
IP-10_N_ sCD40L_Ag_ TGF-α_N_ IFN-γ_Ag-N_	86.0 (49/57)	68.4 (13/19)	81.6 (62/76)	82.5 (47/57)	63.2 (12/19)	0.3612	125.5
IL-1α_Ag_ IP-10_N_ sCD40L_Ag_ IFN-γ_N_	82.5 (47/57)	78.9 (15/19)	81.6 (62/76)	80.7 (46/57)	68.4 (13/19)	0.3514	131.0
IP-10_N_ sCD40L_Ag_ TGF-α_N_ IFN-γ_Ag_	84.2 (48/57	68.4 (13/19)	80.2 (61/76)	80.7 (46/57)	57.9 (11/19)	0.3631	124.5
IP-10_N_ MCP-3_N_ sCD40L_Ag_ IFN-γ_Ag-N_	86.0 (49/57)	73.7 (14/19)	82.9 (63/76)	84.2 (48/57)	63.2 (12/19)	0.3519	130.7
IL-1Ra_Ag_ IP-10_N_ sCD40L_Ag_ IFN-γ_Ag-N_	86.0 (49/57)	68.4 (13/19)	81.6 (62/76)	84.2 (48/57)	57.9 (11/19)	0.302	164.3
IL-1Ra_N_ IP-10_N_ sCD40L_Ag_ IFN-γ_Ag_	87.7 (50/57)	68.4 (13/19)	82.9 (63/76)	86.0 (49/57)	57.9 (11/19)	0.312	156.5

Participants were not stratified according to Quantiferon results or HIV status prior to analysis. In each case, effect df = 1, error df = 71. P- values for all the models were <0.0001. N = unstimulated marker levels, Ag = levels detected in antigen stimulated supernatants, Ag-N = Antigen specific marker levels obtained after background correction.

### Utility of the Markers Investigated in Discriminating between LTBI and Active TB Disease in Quantiferon Positive Children

Some of the markers investigated in this study such as EGF, MIP-1β, IL-1α, TGF-α, sCD40L and VEGF, were shown to have potential in discriminating between *M.tb* infection and active TB disease in adults in a previous study conducted in the same setting [20]. To investigate the potential utility of these markers in children, the levels obtained in QFT-IT positive non TB cases (N = 26) were compared to the levels in the QFT-IT positive children with TB disease (N = 15), regardless of HIV status. When analysis was performed in all the 41 QFT-IT positive study participants, the Mann Whitney U test showed significant differences or trends for the unstimulated, antigen stimulated or antigen-specific levels of six of the 12 markers evaluated, namely EGF, IFN-α2, IL-1Ra, IP-10, sCD40L and VEGF. The median unstimulated levels of IL-1Ra were significantly higher in the *M.tb* infected children while the median unstimulated levels of IP-10 (N), EGF (Ag) and VEGF (Ag-N) were significantly higher in the TB cases (Table 4). After ROC analysis, AUC was ≥0.70 only for IL-1Ra (N), IP-10 (N) and VEGF (Ag-N) levels (Table 4). With the exception of IL-1Ra (N) levels, all the markers with AUC ≥0.70 discriminated between *M.tb* infection and active TB disease with both sensitivity and specificity ≥73.0% using the cut-off values selected according to the highest likelihood ratio (Table 4). The median levels of all the markers investigated, in the children with TB disease, those with LTBI and the *M.tb* un-infected children is shown in Table S1.

**Table 4:** Median levels of analytes (pg/ml) and ranges (in parenthesis), and accuracies in discriminating between active TB disease and LTBI in all QFT-IT positive participants.

Marker	LTBI (n = 26)	TB cases (n = 15)	P-value	AUC (95% CI)	Cut-off	Sensitivity, %(95% CI)	Specificity, % (95% CI)
EGF_Ag_	162.1 (50.2–435.7)	244.9 (17.4–635.3)	0.05	0.69 (0.51–0.86)	>380.2	26.7 (7.8–55.1)	96.2 (80.4–99.9)
IFN-α2_N_	1.8 (0.0–142.5)	0.0 (0.0–109.8)	0.08	0.66 (0.49–0.83)	<4.7	93.3 (68.0–99.8)	42.3 (23.4–63.0)
IFN-α2_Ag_	4.7 (0.0–138.5)	0.0 (0.0–133.2)	0.08	0.66 (0.49–0.84)	<4.7	86.7 (59.5–98.3)	50.0 (29.3–70.1)
**IL-1Ra_N_**	**163.6 (0.0–** **4991.4)**	**52.3 (0.0–717.0)**	**0.03**	**0.71 (0.53–0.89)**	**<11.8**	**33.3 (11.8–61.6)**	**96.2 (80.4–99.9)**
**IP-10_N_**	**3959.7 (991.9–** **>20000)**	**10064.2 (1075.0–** **>20000)**	**0.02**	**0.73 (0.55–0.90)**	**>6768**	**73.3 (44.9–92.2)**	**80.8 (60.7–93.5)**
sCD40L_Ag_	5236.7 (2312.0–>20000)	9769.3 (1296.0–>20000)	0.09	0.65 (0.46–0.86)	>9589	53.3(26.6–78.7)	88.5 (69.9–97.6)
**VEGF_Ag-N_**	**−9.5 (−816.8–** **2870.9)**	**292.9 (−99.6–821.9)**	**0.04**	**0.70 (0.53–0.87)**	**>26.8**	**86.7(59.5–98.3)**	**73.1 (52.2–88.4)**

Participants were not stratified according to HIV status prior to analysis. Only analytes that showed significant differences or trends according to the Mann Whitney U test are shown. Analytes that discriminated between active TB disease and LTBI with AUC ≥0.70 after ROC analysis are highlighted in bold. Cut-off values were determined based on the highest likelihood ratio. Sensitivity and specificity are expressed as a percentage. AUC = Area under the ROC curve, 95% CI = 95% confidence interval.

When the accuracies of the markers in discriminating between TB disease and infection was assessed only in HIV-uninfected children (17 QFT-IT positive children without disease and 12 QFT-IT positive children with disease), the unstimulated, antigen stimulated or antigen-specific levels of 5 markers (IFN-α2, IL-1Ra, VEGF, IP-10 and IFN-γ) showed significant differences or trends between the two groups (Table S4). IFN-α2 (N) and IL-1Ra (N) levels were significantly higher in children with TB infection, whereas IFN-γ (N), IP-10 (N), VEGF (N, Ag and Ag-N) levels were significantly higher in the children with TB disease (Table S4). After ROC analysis, AUC was ≥0.72 for all the markers with significant Mann Whitney U test p values between groups. Although VEGF (N) levels discriminated between TB disease and LTBI with a sensitivity of 100%, specificity was only 77% at the selected cut-off value and only IP-10(N), VEGF(N), VEGF(Ag) and VEGF(Ag-N) discriminated between TB disease and *M.tb* infection with both sensitivity and specificity ≥70.0% (Table S4).

**Figure 2:**
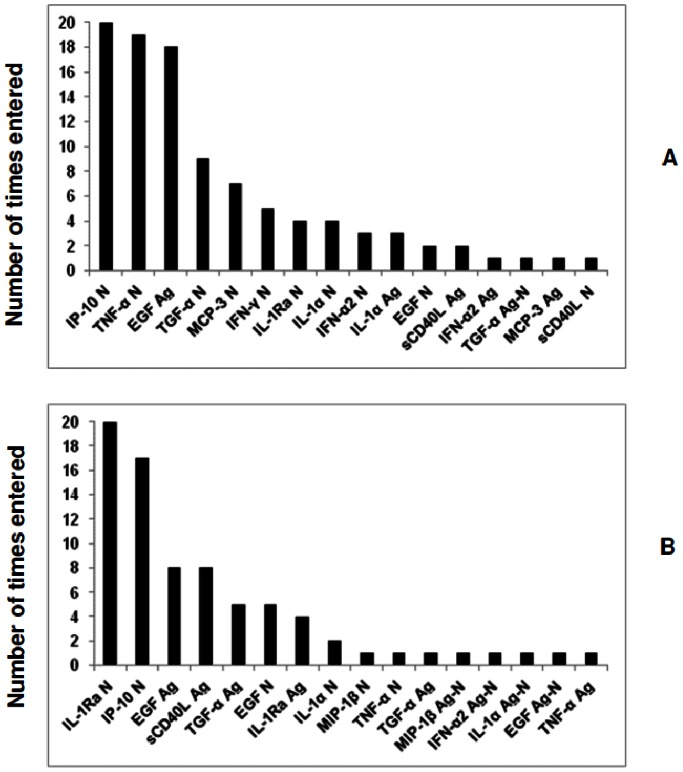
Frequency of analytes in the top 20 GDA models that most accurately classified the Quantiferon positive participants as TB disease (n = 15) or LTBI (n = 39). Participants were not stratified according to HIV infection status prior to analysis of the data. The columns represent the number of times each analyte was included into the top 20 discriminatory models. A = frequency of analytes in the models generated with raw untrimmed data, B = frequency of analytes in models generated after data was trimmed to scale-down the influence of outliers.

When the predictive abilities of combinations of analytes for discriminating between TB disease and *M.tb* infection were assessed by GDA, different 5-analyte combinations predicted up to 81% (21/26) of the *M.tb* infected cases and up to 80% (12/15) of the children with active TB disease after leave-one-out cross validation (Table 5). The predictive accuracy of the marker models increased by using the data trimming procedure for outliers. Different 5-analyte combinations of EGF (Ag), IL-1Ra (N), IP-10 (N), TGF-α (Ag), IL-1α (N), sCD40L (Ag) and IFN-α2 (Ag-N), accurately predicted up to 84.6% (22/26) of the latently infected children and up to 86.7% (13/15) of the QFT-IT positive children with TB disease after leave-one-out cross validation (Table S5). The most frequently occurring analytes in the top 20 GDA models that best predicted infection or TB disease using the raw untrimmed data included IP-10 (N), TNF-α (N) and EGF (Ag) while the most frequently occurring analytes in the top 20 models generated with the trimmed data included IL-1Ra (N), IP-10(N), EGF (Ag) and sCD40L (Ag) (Figure 2).

**Table 5:** General discriminant analysis (GDA) models for discriminating between LTBI and active TB disease.

Analytes	Resubstitution Classification matrix			Leave-one-out Cross validation		Wilks lambda	f
	No TB (%)	TB (%)	Total (%)	No TB (%)	TB (%)		
EGF_Ag_ IL-1Ra_N_ IP-10_N_ MCP-3_N_ TNF-α_N_	80.8 (21/26)	80.0 (12/15)	80.5 (33/41)	80.8 (21/26)	80.0 (12/15)	0.319	74.5
EGF_Ag_ IL-1α_N_ IP-10_N_ MCP-3_N_ TNF-α_N_	88.5 (23/26)	80.0 (12/15)	85.4 (35/41)	80.8 (21/26)	73.3 (11/15)	0.316	75.9
EGF_N_ IL-1α_N_ IP-10_N_ MCP-3_N_ TNF-α_N_	80.8 (21/26)	86.7 (13/15)	82.9 (34/41)	80.8 (21/26)	80.0 (12/15)	0.364	61.0
EGF_Ag_ IP-10_N_ sCD40L_N_ TNF-α_N_TGF-α_N_	76.9 (20/26)	80.0 (12/15)	78.0 (32/41)	73.1 (19/26)	73.3 (11/15)	0.355	63.4
EGF_Ag_ IL-1α_N_ IP-10_N_ TGF-α_N_ TNF-α_N_	80.8 (21/26)	86.7 (13/15)	82.9 (34/41)	76.9 (20/26)	80.0 (12/15)	0.350	64.9
EGF_Ag_ IL-1α_N_ IP-10_N_ TNF-α_N_ IFN-γ_N_	92.3 (24/26)	73.3 (11/15)	85.4 (35/41)	76.9 (20/26)	73.3 (11/15)	0.317	75.4
EGF_N_ IL-1Ra_N_ IP-10_N_ TNF-α_N_ MCP-3_N_	76.9 (20/26)	86.7(13/15)	80.5 (33/41)	76.9 (20/26)	73.3 (11/15)	0.378	57.6

Participants were not stratified according to HIV status prior to data analysis. In each case, effect df = 1, error df = 32. P- values for all the models were <0.0001. N = unstimulated marker levels, Ag = levels detected in antigen stimulated supernatant, Ag-N = Antigen specific marker levels obtained after background correction.

## Discussion

We investigated the diagnostic potential of 12 host markers, detectable in stimulated and unstimulated QFT-IT supernatants, for the diagnosis of TB disease and for discriminating between *M.tb* infection and active TB in 76 children, 22 (29.0%) of whom were HIV-infected. The most promising markers for discriminating between TB disease and no disease in all study participants, regardless of HIV infection status, included IFN-α2 (N and Ag), IL-1Ra (N), sCD40L (N and Ag) and VEGF (N, Ag and Ag-N), whereas IL-1Ra (N), IP-10 (N), and VEGF(Ag-N), showed the most potential in discriminating between *M.tb* infection and active TB. The diagnostic accuracy of the markers increased when markers were used in combinations.

IFN-γ is essential for the control of intracellular pathogens including *M.tb* [34] and it is therefore one of the first markers investigated in most T cell based studies. Although IFN-γ based assays (IGRAs) are now standard for diagnosing *M.tb* infection in some settings [10], these assays do not discriminate between active TB disease and *M.tb* infection [11]. However, ESAT-6 and CFP-10, the main antigens used in these tests, are among the most immunogenic and specific *M.tb* antigens known, even though they are also expressed in some non-tuberculous mycobacteria [35]. The high immunogenicity and relatively high specificity of these antigens for *M.tb*, coupled with the well-established IGRA (especially QFT-IT) platform have contributed to the recent focus on identifying host markers other than IFN-γ, which could discriminate between different *M.tb* infection states in supernatants after stimulation with these antigens. Our work so far indicates that such discriminatory markers may not only be detectable in antigen stimulated, but also in unstimulated supernatants.

The unstimulated, antigen stimulated or antigen-specific levels of 8 of the 12 markers evaluated in the current study (IFN-α2**,** IL-1Ra**,** IP-10**,** sCD40L**,** IFN-γ, VEGF, TGF-α and EGF), were different between children with TB disease and those without disease, and/or between the QFT-IT positive children with disease and those without, irrespective of HIV infection. IFN-α2 is an inflammatory protein that is induced in dendritic cells and monocytes upon infection with *M.tb* [36] and is released by the host as a danger signal, thereby favouring the differentiation of monocytes into dendritic cells [37]. It enhances the production of IP-10, a key chemokine involved in the trafficking of effector TH1 cells to inflammatory sites in vivo [38], by antigen presenting cells [36]. IL-1Ra is an anti-inflammatory protein secreted by various immune cell types including epithelial cells, adipocytes, stromal cells, keratinocytes and hepatocytes. Its levels are elevated in many inflammatory and infectious diseases including TB, and serum levels decline with treatment [39]; its main role is the competitive inhibition of the inflammatory effects of IL-1α and IL-1β [40], [41]. EGF, VEGF and TGF-α are growth factors abundant in pulmonary TB granulomas, including areas of caseous necrosis, and provide good growth environments for mycobacteria [42], [43]. VEGF has been associated with disease activity in both pleural TB and TB meningitis [44], [45] and levels decline after successful TB treatment [46]. CD40L is a co-stimulatory molecule that is expressed on activated CD4+ T cells, and is involved in their activation and development of effector functions [47]. Higher plasma levels of sCD40L have been observed in patients with cavitary TB lesions compared to those without such lesions [48] and the interaction of the CD40-CD40L axis with IFN-γ is important in the generation of giant cells needed for protection in TB and sarcoidosis [49]. All these host markers have previously been evaluated in QFT-IT supernatants and shown to be potentially useful for use singly or in combination with IFN-γ for diagnosing *M.tb* infection (especially IP-10 and TNF-α) [17], [18], [27], [50], [51], or for discriminating between TB disease and LTBI (IP-10, TNF-α, EGF, TGF-α, and VEGF) [16], [19], [20], [28]. Although our findings agree with some of these previous observations, direct comparison of the performances of markers between studies remains difficult because of the different combinations investigated in different studies. In addition, most published studies involved adult participants, and study designs and populations are highly variable.

Kellar et al [18] observed high antigen-specific levels of IFN-γ, IP-10, TNF-α, MIP-1β, MCP-1, IL-2, IL-6 and IL-8 in QFT-IT supernatants from culture confirmed TB cases compared to controls at low risk of infection. The study did not discriminate between TB disease and LTBI. We also observed higher levels of IFN-γ, IP-10 and TNF-α in children with TB disease in the present study; the levels of IL-2, IL-6, IL-8 and MCP-1 were not investigated. There were no significant differences in the levels of MIP-1β and TNF-α between children with TB disease and those without active TB disease (LTBI and uninfected children combined), or between latent *M.tb* infection and active TB disease in this study. However, significant differences were observed when the antigen-specific levels of MIP-1β obtained in the children with active disease or LTBI, were compared to the levels obtained in the uninfected children, therefore agreeing with the observations of Kellar et al for MIP-1β. Although IL-15 and MCP-1 together were the most useful markers for discriminating between TB disease and LTBI, in a study conducted by Frahm et al [17], other markers (IL-1Ra, IFN-α, and IL-4) also showed differences between groups but had limited clinical utility. We found IL-1Ra and IFN-α2 to be amongst the most potentially useful markers for ascertaining TB disease and discriminating between *M.tb* infection and active TB, with higher levels in the children without active TB disease. These observations agree with the study by Frahm et al [17]. Our study population consisted of young children and comparison with adult studies might not be appropriate given that the levels of biomarkers in QFT-IT supernatants have been shown to be different even amongst children of different age groups within the same cohorts [52] and there are vast differences in the immune responses between children and adults. For paediatric studies in which the levels of multiple host markers in QFT-IT supernatants were investigated, similar variations in results have been obtained as observed in adults.

All the markers investigated in the present study were amongst the 29 host markers previously evaluated in QFT-IT supernatants from children in a low TB endemic environment [52]. The one marker that showed differences between LTBI and active TB, IL-2, was not available in the panel investigated in our study. Among the common markers between the current and adult studies conducted in the same community [20], [33], only sCD40L and VEGF responses were promising in univariate analysis in this study (AUC≥0.70), together with novel markers (IFN-α2, IL-1Ra). However, TGF-α, TNF-α and IL-1α each featured in at least one of the top 20 GDA models that accurately classified ≥80% of all enrolled. Larger studies may help to better understand the significance of these markers in children, and explain the differences observed compared to adults. For example, age-related developmental changes in the immune system may play a role and can only be explored in large paediatric studies encompassing a wide age-range. In addition, we did not examine disease severity as a covariate given our limited sample size. The influence of disease severity and other factors which might contribute to findings and which were not investigated in this preliminary study, including the nutritional status of the infant, the timing of presentation and the age of the child should be taken into account in future, larger studies.

Of the 76 children included in our study, 29% were HIV infected. Some of the markers that showed potential in HIV-uninfected adults (EGF and IL-1α) [20], showed similar differences only when analysis was performed in HIV-uninfected children. This might suggest that the performance of at least EGF and IL-1α might be influenced by HIV co-infection. However, because only 4 of the 19 children with TB disease were co-infected witih HIV in this study, we cannot draw strong conclusions on the possible influence of HIV on the performance of the markers. HIV co-infection is common among TB cases in our setting and may complicate diagnosis. It will be important to investigate the influence of HIV on the performance of the markers in future larger studies, especially if the promising accuracy of these markers is maintained in validation studies. More data on the potential markers identified in QFT-IT supernatants to date, especially in children of different age-groups and with different spectrum of disease, is necessary, as there is an urgent need for diagnostic tests tailored for use in this high-risk and diverse patient group. New studies may also help make sense of the inconsistent results obtained in the studies published so far, given the large numbers of markers that are often evaluated and the small study participant numbers.

IGRAs remain well-established for the diagnosis of *M.tb* infection. There is evidence that IP-10 measurement in QFT-IT supernatants may perform similarly or even better [50], [53]. However, diagnostic tests which can discriminate accurately between LTBI and active TB will be valuable in settings with a high proportion of latently infected individuals and limited resources [54]. Novel markers with discriminatory potential between *M.tb* infection and disease could be investigated directly in culture supernatants after overnight incubation of QFT-IT tubes, or used as a rule-in test after IFN-γ or IP-10 detection. In the present study, unstimulated IP-10 levels showed potential in discriminating between active TB and latent infection. We could not assess antigen-specific IP-10 levels because the levels of IP-10 elicited upon stimulation with antigen were above the range of detection of the standard curve in most of the study participants. However other investigators have shown that antigen-specific levels of IP-10 (Ag-N) are not useful in discriminating between *M.tb* infection and active disease both in adults [20], [55], [56] and children [27], [51]. In the present study, higher unstimulated levels of IP-10 were observed in the children with TB disease and this is contrary to what was observed for instance, in the studies by Whittaker et al. [27] and Wang et al. [57]. The reasons for this discrepancy in background levels is unknown, but at least, 25% of the TB cases elicited IP-10 levels which were in the detectable range of the standard curve, compared to the LTBI cases in whom antigen stimulated levels were above the range of the curve in 92% (24/26) of the children investigated (Table S1). This might suggest that the IP-10Ag and IP-10Ag-N levels obtained in this study might have indeed been higher in the LTBI cases as observed in these previous studies.

The most accurate single marker for discriminating between TB disease and no disease or *M.tb* infection and active disease in this study (VEGF), has previously been shown to be potentially useful in serum samples [46]. Future studies should investigate models based on unstimulated, antigen stimulated or antigen-specific responses to determine the best marker/model and under what stimulation condition (unstimulated, antigen stimulated (Ag) or antigen-specific responses (Ag-N)) might be most useful. Any unstimulated marker levels shown to be useful for diagnostic purposes either singly or in combination with other markers or clinical information could be measured directly in serum or plasma, but studies comparing the levels of the marker in these sample types would have to be performed first. Ultimately, immunological biomarker tests will have greatest impact if incorporated into rapid, easy-to-use test platforms such as the lateral flow technology.

The main limitations of our study include the relatively small sample size and the case-control design. It is possible that we might have reported a significant finding which occurred by chance, given that 12 host markers were evaluated. Such a risk occurs in all large biomarker discovery studies regardless of the discovery platform used. A corrective measure that is usually employed during statistical analysis to limit this risk is the correction for multiple comparisons. The main analytical procedure employed in this study was ROC analysis, a method in which no hypothesis testing is done, and decisions resulting from likelihood ratios [58]. Not correcting for multiple comparisons however, may be a concern in GDA as the best subsets method (used in evaluating marker combinations in this study) does generate different analyte combinations. This also, is not a major concern as the focus of this manuscript was not on p-values for a specific combination of analytes but on which analytes occurred most frequently in the multi-marker models.

Future studies should evaluate the host markers in children and adults who are immuno-compromised, in extrapulmonary TB cases, and also in individuals with other lung diseases [59].

### Conclusions

Our findings indicate that multiple host markers detected in QFT-IT supernatants, especially IFN-α2, IL-1Ra, sCD40L, IP-10 and VEGF, have potential to support the diagnosis of TB disease or the discrimination between TB disease and LTBI in children. Our results also indicate that unstimulated host marker levels might be useful and warrant further investigation in larger prospective studies.

## Supporting Information

Table S1 **Median levels (pg/ml) of all host markers (Inter-quartile ranges in parenthesis) in all children with TB diseases, latent** ***M.tb*** **infection or no** ***M.tb*** **infection and p-values for differences between the groups.** Significant p-values are highlighted in bold. Nd = not determined, N = unstimulated marker levels, Ag = levels detected in antigen stimulated supernatant, Ag-N = Antigen-specific marker levels obtained after background correction.
(DOCX)

Table S2 **Median levels of analytes (pg/ml) and ranges (in parenthesis), and accuracies in the diagnosis of TB disease in HIV uninfected children.** Only analytes that showed significant differences or trends according to the Mann Whitney U test are shown. Analytes that discriminated between TB disease and no TB with AUC ≥0.70 after ROC analysis are highlighted in bold. Cut-off values were determined based on the highest likelihood ratio. Sensitivity and specificity are expressed as a percentage. AUC = Area under the ROC curve, 95% CI = 95% confidence interval.
(DOCX)

Table S3 **General discriminant analysis (GDA) models for discriminating between TB disease and no TB.** The influence of outliers was scaled down by trimming the data and the GDA analysis done in all study participants, regardless of HIV infection or QFT-IT results. In each case, effect df = 1, error df = 70. P- values for all the models were <0.0001. N = unstimulated marker levels, Ag = levels detected in antigen stimulated supernatant, Ag-N = Antigen specific marker levels obtained after background correction.
(DOCX)

Table S4 **Median levels of analytes (pg/ml) and ranges (in parenthesis), and abilities to discriminate between TB disease and LTBI in HIV uninfected QFT-IT positive children.** Only analytes that showed significant differences or trends according to the Mann Whitney U test are shown. Cut-off values were determined based on the highest likelihood ratio. Sensitivity and specificity are expressed as a percentage. AUC = Area under the ROC curve, 95% CI = 95% confidence interval.
(DOCX)

Table S5 **General discriminant analysis (GDA) models for discriminating between TB disease and latent** ***M.tb*** **infection.** The top 20 GDA models after the influence of outliers was scaled down by trimming of data in all QFT-IT positive study participants, regardless of HIV infection status are shown. In each case, effect df = 1, error df = 36. P- values for all the models were <0.0001, otherwise stated. N = unstimulated marker levels, Ag = levels detected in antigen stimulated supernatant, Ag-N = Antigen specific marker levels obtained after background correction, ♯ = p value for model was 0.524.
(DOCX)
