# Supplementary material for: Utility of Host Markers Detected in Quantiferon Supernatants for the Diagnosis of Tuberculosis in Children in a High-Burden Setting
Source: PLoS One. 2013 May 15;8(5):e64226. doi: 10.1371/journal.pone.0064226 (PMC3655018; doi:10.1371/journal.pone.0064226)
Supplement: Table S1 — Median levels (pg/ml) of all host markers (Inter-quartile ranges in parenthesis) in all children with TB diseases, latent M.tb infection or no M.tb infection and p-values for differences between the groups. Significant p-values are highlighted in bold. Nd = not determined, N = unstimulated marker levels, Ag = levels detected in antigen stimulated supernatant, Ag-N = Antigen-specific marker levels obtained after background correction. (DOCX) [file pone.0064226.s001.docx]

**Table S1:** **Median levels (pg/ml) of all host markers (Inter-quartile ranges in parenthesis) in all children with TB diseases, latent *M.tb* infection or no *M.tb* infection and p-values for differences between the groups.** Significant p-values are highlighted in bold. Nd= not determined, N = unstimulated marker levels, Ag = levels detected in antigen stimulated supernatant, Ag-N = Antigen-specific marker levels obtained after background correction

| Marker | TB disease (n=19) | LTBI (n=26) | *M.tb* uninfected (n=31) | P-value TB disease vs LTBI | P-value TB diseased vs uninfected | P-value LTBI vs Uninfected |
| --- | --- | --- | --- | --- | --- | --- |
| EGF_N_ | 338.1 (210-423) | 282.3 (172-348) | 277.4 (164-396) | 0.202 | 0.542 | 0.490 |
| EGF_Ag_ | 244.9 (161-318) | 162.1 (105-282) | 225.3 (141-305) | 0.056 | 0.589 | 0.076 |
| EGF_Ag-N_ | -44.5 (-122-57) | -73.1 (-129--25) | -41.1 (-121- -17) | 0.340 | 0.711 | 0.382 |
| IFN- α2_N_ | 0.0 (0-0) | 1.8 (0-10) | 5.8 (0-28) | **0.035** | **0.002** | 0.185 |
| IFN- α2_Ag_ | 0.0 (0-4) | 4.7 (0-14) | 5.8 (0-33) | **0.034** | **0.013** | 0.422 |
| IFN- α2_Ag-N_ | 0.0 (0-0) | 0.0 (0-0) | 0.0 (-5-0) | 0.225 | 0.087 | 0.384 |
| IL-1Ra_N_ | 63.4 (6-219) | 163.6 (74-300) | 200.0 (85-357) | **0.049** | **0.010** | 0.779 |
| IL-1Ra_Ag_ | 334.8 (35.4-1774.3) | 580.7 (204-1133) | 214.0 (134-397) | 0.303 | 0.689 | **0.006** |
| IL-1Ra_Ag-N_ | 60.1(12-1437) | 191.47(48-967) | 29.5(-60-149) | 0.880 | **0.018** | **0.003** |
| IL-1α_N_ | 70.2(19-86) | 50.3(29-138) | 97.5(40-167) | 0.782 | 0.067 | 0.335 |
| IL-1α_Ag_ | 128.6(89-170) | 144.9(105-177) | 107.1(61-198) | 0.388 | 0.453 | 0.129 |
| IL-1α_Ag-N_ | 40.9(16-109) | 62.0(15-103) | 7.4(0-66) | 0.990 | **0.026** | **0.015** |
| IP-10_N_ | 7612.8(4779-12770) | 3959.7(2555-6293) | 4071.1(1716-10943) | **0.029** | 0.086 | 0.929 |
| IP-10_Ag_ | >20000.0(18713->20000) | >20000(>20000->20000) | 7955.3(2831-16916) | nd | nd | nd |
| IP-10_Ag-N_ | nd | nd | 1282.2(118-5281) | nd | nd | nd |
| MCP-3_N_ | 2085.5(636-5621) | 3018.5(1800-5229) | 3883.5(1440-9788) | 0.197 | 0.068 | 0.495 |
| MCP-3_Ag_ | 1447.1(444-5099) | 3303.7(661-9699) | 2881.4(1120-6805) | 0.236 | 0.211 | 0.917 |
| MCP-3_Ag-N_ | -406.7(-733-203.98) | 38.7(-1074-3175) | -606.6(-4239-132) | 0.322 | 0.303 | **0.049** |
| MIP-1β_N_ | 1895.7(901-3148) | 2217.3(1306-3756) | 2136.7(1167.8-4195) | 0.476 | 0.373 | 0.872 |
| MIP-1β_Ag_ | 3627.1(816-10081) | 4487.3(2824-7675) | 2209.3(1238.3-5617) | 0.305 | 0.561 | **0.008** |
| MIP-1β_Ag-N_ | 831.6(-179-7369) | 1714.7(430-3891) | -209.9(-975-997) | 0.557 | **0.045** | **0.002** |
| sCD40L_N_ | 7858.2(5358-13358) | 6283.8(4008-8509) | 3977.6(2916-6053) | 0.126 | **0.002** | **0.023** |
| sCD40L_Ag_ | 9769.3(3751-14129) | 5236.7(3326-7393) | 3575.0(2404-5964) | **0.043** | **0.001** | **0.024** |
| sCD40L_Ag-N_ | 1147.2(-3931-5975.9) | -503.7(-1472-766) | -387.0(-1514-516) | 0.167 | 0.073 | 0.980 |
| TGF- α_N_ | 7.9(5-14) | 9.5(6-14) | 10.1(7-21) | 0.636 | 0.192 | 0.399 |
| TGF- α_Ag_ | 7.9(7-23) | 11.8(7-17) | 10.4(6-19) | 0.356 | 0.817 | 0.596 |
| TGF- α_Ag-N_ | 3.3(-1-7) | 2.3(-4-8) | -1.4(-7-4) | 0.637 | **0.046** | 0.191 |
| TNF- α_N_ | 207.8(95-359) | 136.4(65-244) | 119.6(58-215) | 0.357 | 0.246 | 0.860 |
| TNF- α_Ag_ | 257(46-494) | 111.5(68-334) | 97.3(45-208) | 0.747 | 0.207 | 0.290 |
| TNF-α_Ag-N_ | 34(-77-117) | -14.6(-60-87) | -15.0(-83-27) | 0.504 | 0.200 | 0.730 |
| VEGF_N_ | 727.5(649-823) | 624.5(241-992) | 412.6(0-748) | 0.549 | **0.008** | 0.141 |
| VEGF_Ag_ | 963.7(823-1162) | 741(0-1110) | 369.4(0-840) | 0.137 | **<0.001** | 0.102 |
| VEGF_Ag-N_ | 302.4(75-456) | -9.5(-348-432) | 0.0(-151-184) | **0.025** | **0.010** | 0.686 |
| IFN-γ_N_ | 11.2(1-50) | 11.7(0.9-26) | 10.0(3-26) | 0.555 | 0.710 | 0.993 |
| IFN-γ_Ag_ | 196.3(57-1297) | 594.5(261-1183) | 11.2(4-23) | 0.151 | **<0.001** | **<0.001** |
| IFN-γ_Ag-N_ | 170.4(18-1310) | 568.6(212-1158) | 0.0(-2-4) | 0.184 | **<0.001** | **<0.001** |
