# Supplementary material for: Utility of Host Markers Detected in Quantiferon Supernatants for the Diagnosis of Tuberculosis in Children in a High-Burden Setting
Source: PLoS One. 2013 May 15;8(5):e64226. doi: 10.1371/journal.pone.0064226 (PMC3655018; doi:10.1371/journal.pone.0064226)
Supplement: Table S2 — Median levels of analytes (pg/ml) and ranges (in parenthesis), and accuracies in the diagnosis of TB disease in HIV uninfected children. Only analytes that showed significant differences or trends according to the Mann Whitney U test are shown. Analytes that discriminated between TB disease and no TB with AUC ≥0.70 after ROC analysis are highlighted in bold. Cut-off values were determined based on the highest likelihood ratio. Sensitivity and specificity are expressed as a percentage. AUC = Area under the ROC curve, 95% CI = 95% confidence interval. (DOCX) [file pone.0064226.s002.docx]

**Table S2: Median levels of analytes (pg/ml) and ranges (in parenthesis), and accuracies in the diagnosis of TB disease in HIV uninfected children**. Only analytes that showed significant differences or trends according to the Mann Whitney U test are shown. Analytes that discriminated between TB disease and no TB with AUC ≥ 0.70 after ROC analysis are highlighted in bold. Sensitivity and specificity are expressed as a percentage. AUC = Area under the ROC curve, 95% CI = 95% confidence interval.

| Marker | No TB (n=39) | TB (n=15) | P-value | AUC(95% CI) | Cut off | Sensitivity, % (95% CI) | Specificity, % (95% CI) |
| --- | --- | --- | --- | --- | --- | --- | --- |
| **IFN-α2_N_** | **5.8 (0.0-299.4)** | **0.0 (0.0-109.8)** | **0.0028** | **0.75 (0.60-0.89)** | **<1.8** | **86.7 (59.5-98.3)** | **64.1(47.2-78.8)** |
| **IFN-α2_Ag_** | **5.8 (0.0-269.4)** | **0.0 (0.0-133.2)** | **0.0061** | **0.72 (0.57-0.87)** | **<1.8** | **86.7 (59.5-98.3)** | **59.0(42.1-74.4)** |
| **IL-1Ra_N_** | **208.0 (0.0-4991.4)** | **52.3 (0.0-717.0)** | **0.014** | **0.72 (0.55-0.89)** | **<11.8** | **33.3(11.8-61.6)** | **97.4(86.5-99.9)** |
| IP-10_N_ | 2864.0(474.6->20000) | 7612.8 (1075.0->20000) | 0.031 | 0.69(0.52-0.85) | >6878 | 60(32.3-83.6) | 82.0(66.5-92.5) |
| sCD40L_N_ | 5396.4(1571.7->20000) | 8399.1(1177.2->20000) | 0.02 | 0.69(0.51-0.87) | >10541 | 46.7(21.3-73.4) | 92.3(79.1-98.4) |
| **VEGF_N_** | **240.5(0.0-1939.5)** | **747.5 (0.0-1011.7)** | **0.0014** | **0.79(0.65-0.93)** | **>631.3** | **85.7(57.2-98.2)** | **82.0(66.5-92.5)** |
| **VEGF_Ag_** | **55.8(0.0-2870.9)** | **963.7 (325.4-1741.8)** | **0.00014** | **0.84(0.73-0.95)** | **>821.7** | **80.0(51.9-95.7)** | **84.6(69.5-94.1)** |
| **VEGF_Ag-N_** | **0.0 (0.0-2870.9)** | **302.4 (0-821.9)** | **0.0086** | **0.73(0.59-0.87)** | **>52.0** | **80.0(51.9-95.7)** | **76.9(60.7-88.9)** |
| IFN-γ_Ag_ | 32.8 (0.0-8774.9) | 196.3 (5.2-5564.3) | 0.037 | 0.68(0.54-0.84) | >2623 | 26.7(7.8-55.1) | 97.4(86.5-99.9) |
| **IFN-γ_Ag-N_** | **13.1(0.0-8774.9)** | **170.4(0.0-5564.3)** | **0.019** | **0.70(0.57-0.85)** | **>23.6** | **80.0(51.9-95.7)** | **59.0(42.1-74.4)** |
| EGF_N_ | 247.9 (41.3-904.1) | 333.8(110.9-602.3) | 0.096 | 0.65(0.48-0.81) | >418.3 | 26.7(7.8-55.1) | 89.7(75.8-97.1) |
| IL-1α_Ag-Nil_ | 0.0 (0.0-44.0) | 40.9 (3.1-309.1) | 0.07 | 0.66(0.50-0.81) | >235.5 | 26.7(7.8-55.1) | 97.4(86.5-99.9) |
| sCD40L_Ag_ | 4277.0 (1135.0->20000) | 10424.4 (1296.0->20000) | 0.051 | 0.67(0.48-0.86) | >9671 | 60.0(32.3-83.6) | 89.7(75.8-97.1) |
