# Supplementary material for: Utility of Host Markers Detected in Quantiferon Supernatants for the Diagnosis of Tuberculosis in Children in a High-Burden Setting
Source: PLoS One. 2013 May 15;8(5):e64226. doi: 10.1371/journal.pone.0064226 (PMC3655018; doi:10.1371/journal.pone.0064226)
Supplement: Table S3 — General discriminant analysis (GDA) models for discriminating between TB disease and no TB. The influence of outliers was scaled down by trimming the data and the GDA analysis done in all study participants, regardless of HIV infection or QFT-IT results. In each case, effect df = 1, error df = 70. P- values for all the models were <0.0001. N = unstimulated marker levels, Ag = levels detected in antigen stimulated supernatant, Ag-N = Antigen specific marker levels obtained after background correction. (DOCX) [file pone.0064226.s003.docx]

**Table S3:** **General discriminant analysis (GDA) models for discriminating between TB disease and no TB**. The influence of outliers was scaled down by trimming the data and the GDA analysis done in all study participants, regardless of HIV infection or QFT results. In each case, effect df =1, error df = 70. P- Values for all the models were < 0.0001. N = unstimulated marker levels, Ag = levels detected in antigen stimulated supernatant, Ag-N = Antigen specific marker levels obtained after background correction.

| Analytes | Resubstitution Classification matrix | | | Leave-one-out Cross validation | | Wilks lambda | f |
| --- | --- | --- | --- | --- | --- | --- | --- |
|  | No TB (%) | TB (%) | Total (%) | No TB (%) | TB (%) |  |  |
| IFN-α2_Ag_ IP-10_N_ IL-1Ra_N_ sCD40L_Ag_ TGF-α_Ag_ | 82.5 (47/57) | 89.5 (17/19) | 84.2 (64/76) | 80.7 (46/57) | 73.7 (14/19) | 0.534 | 61.0 |
| IFN-α2_N_ IL-1Ra_N_ IP-10_N_ sCD40L_Ag_ TGF-α_Ag-N_ | 84.2 (48/57 | 84.2 (16/19) | 84.2 (64/76) | 78.9 (45/57) | 73.7 (14/19) | 0..512 | 66.8 |
| IL-1Ra_N_ IL-1α_N_ IP-10_N_ sCD40L_Ag_ TGF-α_Ag-N_ | 84.2 (48/57) | 89.5 (17/19) | 85.5 (65/76) | 82.5 (47/57)) | 84.2 (16/19) | 0.476 | 77.1 |
| IFN-α2_Ag_ IL-1Ra_N_ IL-1ra_Ag_ IP-10_N_ sCD40L_Ag_ | 84.2 (48/57) | 78.9 (15/19) | 82.9 (63/76) | 84.2 (48/47) | 73.7 (14/19) | 0.480 | 75.7 |
| IFN-α2_Ag_ IL-1Ra_N_ IP-10_N_ sCD40L_Ag_ TNF-α_Ag_ | 86.0 (49/57) | 84.2 (16/19) | 85.5 (65/76) | 82.5 (47/57) | 73.7 (14/19) | 0.467 | 79.8 |
| IFN-α2_N_ IL-1Ra_N_ IL-1Ra_Ag_ IP-10_N_ sCD40L_Ag_ | 84.2 (48/57) | 78.9 (15/19) | 82.9 (63/76) | 84.2 (48/47) | 73.7 (14/19) | 0.467 | 79.9 |
| IL-1Ra_N_ IL-1Ra_Ag_ IP-10_N_ sCD40L_Ag_ TGF-α_Ag-N_ | 86.0 (49/57) | 78.9 (15/19) | 84.2 (64/76) | 80.7 (46/57) | 68.4 (13/19) | 0.0.426 | 94.5 |
| IL-1Ra_N_ IL-1Ra_Ag_ IP-10_N_ sCD40L_Ag_ TGF-α_Ag-N_ | 86.0 (49/57) | 84.2 (16/19) | 85.5 (65/76) | 82.5 (47/57) | 73.7 (14/19) | 0.422 | 95.8 |
| IFN-α2_N_ IL-1Ra_N_ IP-10_N_ sCD40L_Ag_ TNF-α_Ag_ | 84.2 (48/57) | 84.2 (16/19) | 84.2 (64/76) | 82.5 (47/57) | 73.7 (14/19) | 0.458 | 82.9 |
| IL-1Ra_N_ IP-10_N_ sCD40L_Ag_ TGF-α_Ag-N_ TNF-α_Ag_ | 86.0 (49/57) | 84.2 (16/19) | 85.5 (65/76) | 82.5 (47/57) | 73.7 (14/19) | 0.419 | 97.0 |
| IL-1Ra_N_ IL-1α2_N_ IP-10_N_ sCD40L_Ag_ TNF-α_Ag_ | 86.0 (49/57) | 89.5 (17/19) | 86.8 (66/76) | 84.2 (48/47) | 68.4 (13/19) | 0.430 | 92.6 |
| IFN-α2_Ag_ IP-10_N_ sCD40L_Ag_ IL-1Ra_N_ IL-1Ra_Ag_ | 84.2 (48/57) | 78.9 (15/19) | 82.9 (63/76) | 82.5 (47/57) | 78.9 (15/19) | 0.478 | 76.4 |
| IFN-α2_Ag_ IL-1Ra_N_ IL-1Ra_Ag_ IP-10_N_ TGF-α_Ag-N_ | 80.7 (46/57) | 73.7 (14/19) | 78.9 (60/76) | 78.9 (45/57) | 73.7 (14/19) | 0.559 | 55.2 |
| IFN-α2_N_ IL-1Ra_N_ IL-1Ra_Ag_ IP-10_N_ sCD40L_N_ | 86.0 (49/57) | 78.9 (15/19) | 84.2 (64/76) | 84.2 (48/47) | 78.9 (15/19) | 0.475 | 77.3 |
| IL-1Ra_N_ IL-1Ra_Ag_ IL-1α_N_ IP-10_N_ sCD40L_Ag_ | 86.0 (49/57) | 78.9 (15/19) | 84.2 (64/76) | 84.2 (48/47) | 73.7 (14/19) | 0.441 | 88.6 |
| IFN-α2_Ag_ IL-1Ra_N_ IL-1Ra_Ag_ IP-10_N_ sCD40L_N_ | 84.2 (48/57) | 78.9 (15/19) | 82.9 (63/76) | 82.5 (47/57) | 78.9 (15/19) | 0.494 | 71.7 |
| IL-1Ra_N_ IL-1α_Ag-N_ IP-10_N_ sCD40L_Ag_ TGF-α_Ag-N_ | 87.7 (50/57) | 78.9 (15/19) | 85.5 (65/76) | 82.5 (47/57) | 78.9 (15/19) | 0.424 | 95.0 |
| IL-1Ra_N_ IL-1Ra_Ag_ IL-1α_N_ IP-10_N_ sCD40L_N_ | 84.2 (48/57) | 78.9 (15/19) | 82.9 (63/76) | 82.5 (47/57) | 73.7 (14/19) | 0.438 | 89.7 |
| IFN-α2_N_ IL-1Ra_N_ IL-1Ra_Ag-N_ IP-10_N_ sCD40L_Ag_ | 84.2 (48/57) | 78.9 (15/19) | 82.9 (63/76) | 82.5 (47/57) | 78.9 (15/19) | 0.466 | 80.4 |
| IL-1Ra_N_ IL-1α_N_ IP-10_N_ sCD40L_Ag_ TNF-α_N_ | 86.0 (49/57) | 89.5 (17/19) | 86.8 (66/76) | 84.2 (48/47) | 84.2 (16/19) | 0.445 | 87.2 |
