# Supplementary material for: Utility of Host Markers Detected in Quantiferon Supernatants for the Diagnosis of Tuberculosis in Children in a High-Burden Setting
Source: PLoS One. 2013 May 15;8(5):e64226. doi: 10.1371/journal.pone.0064226 (PMC3655018; doi:10.1371/journal.pone.0064226)
Supplement: Table S4 — Median levels of analytes (pg/ml) and ranges (in parenthesis), and abilities to discriminate between TB disease and LTBI in HIV uninfected QFT-IT positive children. Only analytes that showed significant differences or trends according to the Mann Whitney U test are shown. Cut-off values were determined based on the highest likelihood ratio. Sensitivity and specificity are expressed as a percentage. AUC = Area under the ROC curve, 95% CI = 95% confidence interval. (DOCX) [file pone.0064226.s004.docx]

**Table S4: Median levels of analytes (pg/ml) and ranges (in parenthesis), and abilities to discriminate between TB disease and LTBI in HIV uninfected QFT-IT positive children**. Only analytes that showed significant differences or trends according to the Mann Whitney U test are shown. Cut-off levels were determined based on the highest likelihood ratio. Sensitivity and specificity are expressed as a percentage. AUC = Area under the ROC curve, 95% CI = 95% confidence interval.

| Marker | LTBI group (n=17) | TB group (n=12) | P value | AUC | Cut off | Sensitivity, % (95% CI) | Specificity, % (95% CI) |
| --- | --- | --- | --- | --- | --- | --- | --- |
| IFN-α2_N_ | 5.8 (0.0-142.5 | 0.0 (0.0-109.8) | 0.05 | 0.73 (0.54-0.92) | <1.8 | 91.7 (61.5-99.8) | 58.8 (32.9-81.6) |
| IFN-α2_Ag_ | 0.0 (0.0-138.5) | 0.0 (0.0-133.2) | 0.06 | 0.76 (0.57-0.95) | <1.8 | 91.7 (61.5-99.8) | 64.7 (38.3-85.8) |
| IL-1Ra_N_ | 252.1 (0.0-4991.0) | 29.6 (0.0-717.0) | 0.03 | 0.74 (0.54-0.94) | <52.8 | 66.7 (34.9-90.1) | 88.2 (63.6-98.5) |
| IP-10_N_ | 3619 (991.9-21000) | 8938 (1075-21000) | 0.02 | 0.74 (0.54-0.94) | >4725 | 75.0 (42.8-94.5) | 70.6 (44.0-89.7) |
| VEGF_N_ | 389.2 (0.0-1939) | 767.5 (611.8-977.8) | 0.02 | 0.77 (0.57-0.97) | >577.1 | 100 (71.5-100) | 76.5 (50.1-93.2) |
| VEGF_Ag_ | 407.8 (0.0-2871.0) | 963.7 (584.8-1742.0) | 0.04 | 0.73 (0.54-0.92) | >821.7 | 83.3 (51.6-97.9) | 70.6 (44.0-89.7) |
| VEGF_Ag-N_ | 0.0 (-816.8-2871.0) | 297.6 (-99.61-821.9) | 0.05 | 0.72 (0.52-0.92) | >44.6 | 83.3 (51.6-97.9) | 76.5 (50.1-93.2) |
| IFN-γ_N_ | 7.2 (0.0-49.0) | 30.24 (0.0-83.8) | 0.03 | 0.74 (0.55-0.93) | >14.3 | 66.7 (34.9-90.1) | 70.6 (44.0-89.7) |
