# Supplementary material for: Utility of Host Markers Detected in Quantiferon Supernatants for the Diagnosis of Tuberculosis in Children in a High-Burden Setting
Source: PLoS One. 2013 May 15;8(5):e64226. doi: 10.1371/journal.pone.0064226 (PMC3655018; doi:10.1371/journal.pone.0064226)
Supplement: Table S5 — General discriminant analysis (GDA) models for discriminating between TB disease and latent M.tb infection. The top 20 GDA models after the influence of outliers was scaled down by trimming of data in all QFT-IT positive study participants, regardless of HIV infection status are shown. In each case, effect df = 1, error df = 36. P- values for all the models were <0.0001, otherwise stated. N = unstimulated marker levels, Ag = levels detected in antigen stimulated supernatant, Ag-N = Antigen specific marker levels obtained after background correction, ♯ = p value for model was 0.524. (DOCX) [file pone.0064226.s005.docx]

**Table S5:** **General discriminant analysis (GDA) models for discriminating between TB disease and latent *M.tb* infection.** The top 20 GDA models after the influence of outliers was scaled down by trimming of data in all QFT-IT positive study participants, regardless of HIV infection status are shown. In each case, effect df =1, error df = 36. P- values for all the models were < 0.0001. N = unstimulated marker levels, Ag = levels detected in antigen stimulated supernatant, Ag-N = Antigen specific marker levels obtained after background correction.

| Analytes | Resubstitution Classification matrix | | | Leave-one-out Cross validation | | Wilks lambda | f |
| --- | --- | --- | --- | --- | --- | --- | --- |
|  | LTBI (%) | TB (%) | Total (%) | LTBI (%) | TB (%) |  |  |
| EGF_Ag_ IP-10_N_ IL-1Ra_N_ TGF-α_Ag-N_ | 73.1 (19/26) | 93.3 (14/15) | 80.5 (33/41) | 69.2 (18/26) | 86.7 (13/15) | 0.454 | 43.3 |
| EGF_N_ IL-1Ra_N_ IP-10_N_ TGF-α_Ag-N_ | 76.9 (20/26) | 86.7 (13/15) | 80.5 (33/41) | 73.1 (19/26) | 80.0 (12/15) | 0.483 | 38.5 |
| EGF_Ag_ IL-1Ra_N_ IP-10_N_ TGF-α_Ag-N_ | 80.8 (21/26) | 86.7 (13/15) | 82.9 (34/41) | 69.2 (18/26) | 80.0 (12/15) | 0.864 | 5.7 |
| EGF_Ag_ IL-1Ra_N_ IP-10_N_ MIP-1β_N_ | 76.9 (20/26) | 86.7 (13/15) | 80.5 (33/41) | 69.2 (18/26) | 80.0 (12/15) | 0.471 | 40.4 |
| IL-1Ra_N_ IP-10_N_ sCD40L_Ag_ TGF-α_Ag-N_ | 76.9 (20/26) | 80.0 (12/15) | 78.0 (32/41) | 76.9 (20/26) | 80.0 (12/15) | 0.466 | 41.2 |
| EGF_Ag_ IL-1Ra_N_ IP-10_N_ TNF-α_N_ | 80.8 (21/26) | 86.7 (13/15) | 82.9 (34/41) | 76.9 (20/26) | 80.0 (12/15) | 0.458 | 42.6 |
| EGF_Ag_ IL-1Ra_N_ IP-10_N_ TGF-α_Ag_ | 80.8 (21/26) | 86.7 (13/15) | 82.9 (34/41) | 80.8 (21/26) | 86.7 (13/15) | 0.497 | 36.8 |
| EGF_N_ IL-1Ra_N_ IL-1Ra_Ag_ IP-10_N_ | 76.9 (20/26) | 80.0 (12/15) | 78.0 (32/41) | 76.9 (20/26) | 73.3 (11/15) | 0.475 | 39.7 |
| EGF_Ag_ IL-1Ra_N_ IP-10_N_ sCD40L_Ag_ | 73.1 (19/26) | 80.0 (12/15) | 75.6 (31/41) | 73.1 (19/26) | 66.7 (10/15) | 0.458 | 42.6 |
| EGF_Ag_ IL-1Ra_N_ IL-1Ra_Ag_ IP-10_N_ | 80.8 (21/26) | 73.3 (11/15) | 78.0 (32/41) | 73.1 (19/26) | 66.7 (10/15) | 0.474 | 39.9 |
| IL-1Ra_N_ IL-1Ra_Ag_ IP-10_N_ sCD40L_Ag_ | 76.9 (20/26) | 73.3 (11/15) | 75.6 (31/41) | 76.9 (20/26) | 73.3 (11/15) | 0.433 | 47.1 |
| IL-1Ra_N_ IL-1α_N_ IP-10_N_ sCD40L_Ag_ | 88.5 (23/26) | 86.7 (13/15) | 87.8 (36/41) | 84.6 (22/26) | 80.0 (12/15) | 0.467 | 41.2 |
| IFN-α2_Ag-N_ IL-1Ra_N_ IP-10_N_ sCD40L_Ag_ | 84.6 (22/26) | 80.0 (12/15) | 82.9 (34/41) | 84.6 (22/26) | 80.0 (12/15) | 0.439 | 46.0 |
| EGF_N_ IL-1Ra_N_ IP-10_N_ sCD40L_N_ | 76.9 (20/26) | 86.7 (13/15) | 80.5 (33/41) | 73.1 (19/26) | 80.0 (12/15) | 0.471 | 40.5 |
| IL-1Ra_N_ IL-1α_Ag-N_ IP-10_N_ sCD40L_Ag_ | 80.8 (21/26) | 80.0 (12/15) | 80.5 (33/41) | 80.8 (21/26) | 80.0 (12/15) | 0.441 | 45.7 |
| EGF_N_ EGF_Ag-N_ IL-1Ra_N_ IP-10_N_ | 76.9 (20/26) | 80.0 (12/15) | 78.0 (32/41) | 76.9 (20/26) | 73.3 (11/15) | 0.517 | 33.7 |
| IL-1Ra_N_ IP-10_N_ sCD40L_Ag_ TNF-α_Ag_ | 84.6 (22/26) | 80.0 (12/15) | 82.9 (34/41) | 84.6 (22/26) | 66.7 (10/15) | 0.423 | 49.1 |
| EGF_Ag_ IL-1Ra_N_ IL-1α_N_ IP-10_N_ | 80.8 (21/26) | 80.0 (12/15) | 80.5 (33/41) | 73.1 (19/26) | 80.0 (12/15) | 0.51 | 34.1 |
